# A Phenominological Qualitative Study of Factors Influencing the Migration of South African Anaesthetists

**DOI:** 10.3390/healthcare10112165

**Published:** 2022-10-29

**Authors:** Leilanie Fletcher-Nkile, Busisiwe Mrara, Olanrewaju Oladimeji

**Affiliations:** 1 Anaesthesiology and Critical Care, Faculty of Health Sciences, Walter Sisulu University, Mthatha 5099, South Africa; 2 Department of Public Health, Faculty of Health Sciences, Walter Sisulu University, Mthatha 5099, South Africa

**Keywords:** migration, South African, anaesthetists, phenomenological, qualitative study

## Abstract

Background: The anaesthetic workforce is a scarce resource in South Africa (SA), and the media frequently reports that anaesthetists are leaving the country in search of better opportunities in well-resourced countries. The aim of this study was to explore the factors influencing the migration intentions of South African anaesthetists. Methods: This study utilised a qualitative methodology in the form of virtual interviews. A total of 23 interviews were conducted on purposefully selected participants using a snowball approach. The interviews were transcribed and coded into emerging themes using MAXQDA version 2022. Thematic content analysis was subsequently performed. Results: The study found that all SA-based participants were considering emigrating, while those who had already emigrated had no plans to return to SA in the near future. Push factors prompted the consideration of emigration, and these were related to unsatisfactory living and working conditions in South Africa, such as a high level of crime and corruption, and the country’s overall poor resources and infrastructure. Destination countries were chosen based on their pull factors such as better working conditions and opportunities for professional growth. These pull factors frequently outweighed South Africa’s push factors and are therefore critical in the decision to emigrate. The main recommendations from the participants include facilitating collaboration between the public and private sectors, expanding the available sub-specialties in SA, and recognising fellowships in South African hospitals. The study participants were of the view that South African anaesthesiologists would be motivated to stay by a combination of patriotism and hope for the future. Conclusions and Policy Recommendations: The Anaesthetic workforce in South Africa is at critical risk and is unlikely to stabilise soon. Concerted efforts should be made by all concerned to explore ways of retaining the staff, considering the recommendations of the participants. Relevant key stakeholders in training and regulation of anaesthetics practice such as the Health Professions Council of South Africa, South African Society of Anaesthesiologists, and the Colleges of Medicine of South Africa should collaborate and prioritise mechanisms of monitoring emigration and intervening on modifiable professional and socio-political factors.

## 1. Introduction

There is a global workforce shortage in anaesthesia and surgical disciplines, and South Africa is not immune to this reality [1]. Due to the increased demand for their coveted skills, this global shortage has been a driving factor in the medical migration of health workers from low- and middle-income- to high-income countries [2]. SA has been identified as the epicentre of African migration because it is the primary supplier of health workers to high-income countries [3].

The brain drain from South Africa (SA) results in the loss of critical human resources, putting additional strain on the healthcare sector [4]. The strain is further exacerbated by unequal distribution of resources between the public and private sectors in SA. The public sector employs only a -third of registered doctors in SA, despite serving most of the population [5].

The anaesthetics work force is also critically affected by the staff shortage. According to the South African Society of Anaesthesiologists (SASA), there are 2826 specialist anaesthesiologists in South Africa, implying a density of 4.71 per 100,000 people. This is slightly less than the recommended minimum of 5 anaesthesiologists per 100,000 people [6]. The remainder of the anaesthetics work forceis made up by general practitioner (GP) anaesthetists, resulting in 16.8 per 100,000 population density. Most GP anaesthetists have a Diploma in Anaesthesia ((DA)SA) qualification. They play a vital role in the South African healthcare sector and are also of interest in the emigration discussion.

It has been widely reported that a doctor’s decision to emigrate is influenced more by push factors from the country of origin than by pull factors from the destination country [7]. As a result, a better understanding of the migration intentions and motivations of anaesthetists will assist South African health authorities in improving their recruitment and retention strategies, as well as health system planning.

The migration of health care workers from South Africa has been thoroughly researched, and the crisis has been highlighted; the negative consequences for the donor country cannot be overlooked. However, the factors that influence migration among the SA anaesthetic workforce have not been studied. This study aimed to explore the factors influencing South African anaesthetists’ migration intentions and migration.

## 2. Methods

### 2.1. Study Design

This study used a qualitative design with phenomenological approach and snowballing process for virtual informant interviews. The phenomenological approach is an inquiry strategy in which the researcher seeks to understand the lived experiences of the participants in relation to a specific phenomenon [8].

### 2.2. Study Setting and Population

The research was conducted on South African anaesthesiologists and GP anaesthetists working in the public and private sectors, in South Africa and abroad.

### 2.3. Exclusion and Inclusion Criteria

#### 2.3.1. Inclusion Criteria

1. South African anaesthesiologists with a Master’s in Medicine (MMed) in Anaesthesiology and/or a Fellowship in Anaesthesiology.

2. South African General Practitioner anaesthetists with a Diploma in Anaesthesia.

3. South African GP anaesthetists and anaesthesiologists who have emigrated from South Africa in the previous10 years.

#### 2.3.2. Exclusion Criteria

1. Non-South African anaesthesiologists and GP anaesthetists working in SA

2. South African anaesthesiologists and general practitioner anaesthetists, practicing anaesthetics for <25% of their work.

### 2.4. Sampling Process and Participant’s Recruitment Strategy

This study combined purposive and snowball sampling with a phenomenological approach, commonly practised strategies in qualitative research. This method enabled the researcher to select participants who would be able to share detailed, rich data on the phenomenon under investigation [8].

A total of 23 participants were enrolled and interviewed as the point of saturation was reached, and time constraints were also a limiting factor. The recruitment process entailed contacting potential participants through professional anaesthetic networks throughout South Africa and abroad. Potential participants were contacted via email or WhatsApp^®^ messaging service depending on the contact information provided. A generic message about the study was sent out, inviting potential participants to participate. Further communication with potential participants began only after the participant consented to the interview. Some challenges the researcher faced during recruitment and enrolment included poorresponse on the first invitation and the need to follow up with multiple reminders to confirm interview times with participants. The recruitment, enrolment and data collection occurred simultaneously during the months of July, August, and September 2021.

### 2.5. Data Collection Process

**Figure 1:**
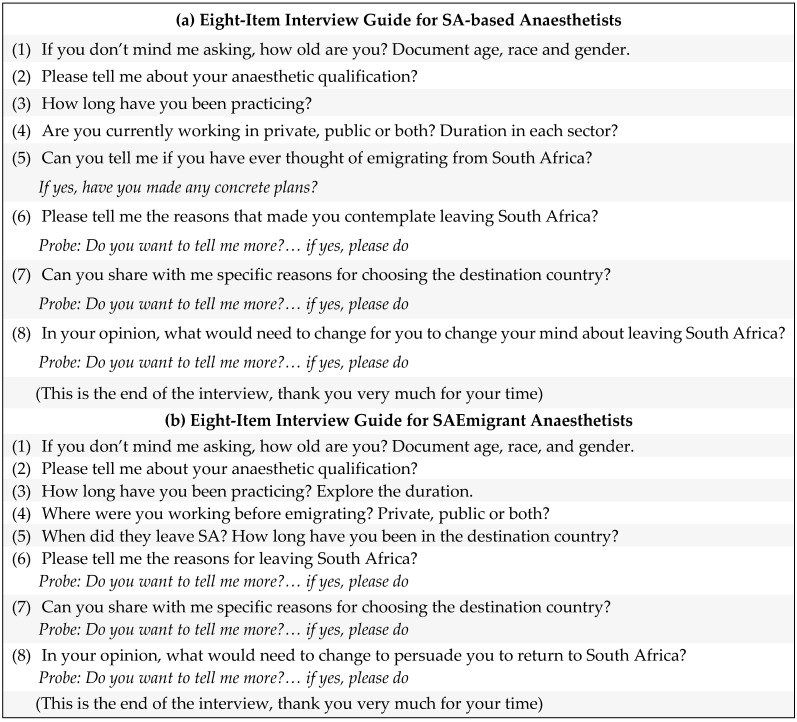
(**a**) Eight-item interview guide for SA-based anaesthetists. (**b**) Eight-item interview guide for South African emigrant anaesthetists.

An eight-item interview guide was used to collect data (as detailed in Figure 1a,b). The interview guide was created using the research questions, and balanced the need for structure and free-flowing conversation.

The interviews were conducted via Zoom^®^, a videoconference call platform. Other platforms, such as Microsoft Teams^®^ and Google Meet^®^, were also available to participants. Participants were all familiar with Zoom^®^, which was the platform of choice for the researcher. After obtaining consent from the participants, the interviews were recorded. During the interview, field notes were taken, especially when internet connectivity was poor. Dropbox was used to save the recordings. The Dropbox files were only accessible for the initial verbatim transcription and subsequent intelligent transcription.

## 3. Data Analysis

The data was analysed using qualitative data analysis (QDA) software MAXQDA version 2022. Data analysis involved thematic analysis of the qualitative data [9].

## 4. Results

### 4.1. Demographics of the Study Participants

**Figure 2:**
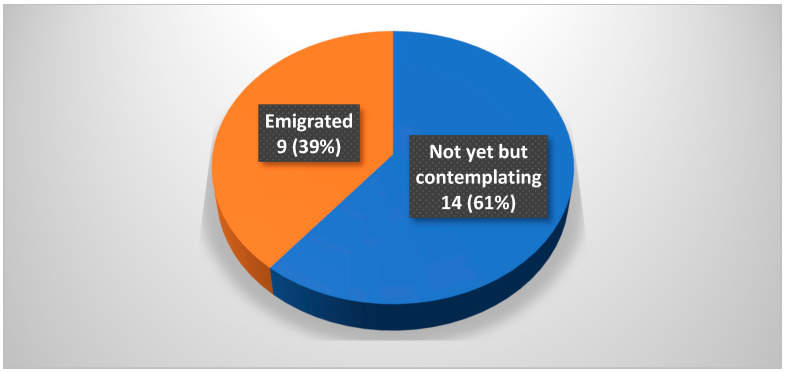
Emigration status of the study participants (n = 23).

**Figure 3:**
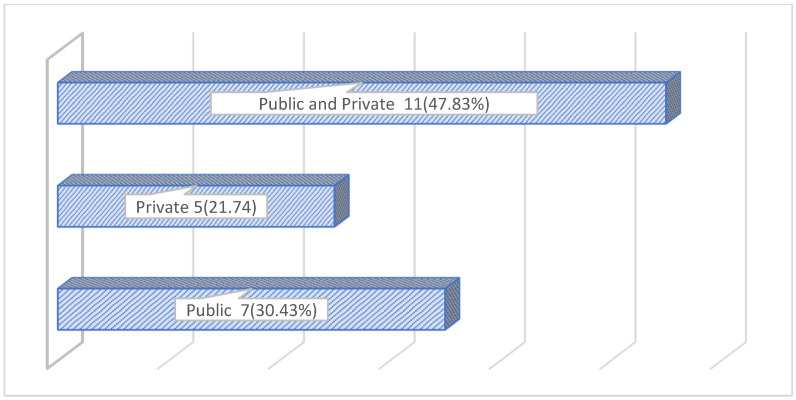
Labour sector of the study participants (n = 23).

The demographic data of the participants are summarised in Table 1. Most participants were aged between 31 and 40 years; 39% of participants had already emigrated while the remaining 61% of participants were based in South Africa (Figure 2). All South African based anaesthetists were contemplating emigration. Most of the participants had 6–10 years of anaesthetic experience, working in both the private and public sectors (Figure 3).

### 4.2. Emerging Themes and Sub-Themes

The qualitative data collected during the interviews with the participants were interpreted by identifying themes and concepts related to each research question. Three main themes emerged, namely, emigration plan, factors influencing the emigration plan and potential factors that would influence the decision not to emigrate, as listed in Table 2.

### 4.3. Emigration Plan

Emigration often starts with contemplation, which leads to making plans to leave, but what ultimately leads to emigration is actually putting these plans into action. This concept is well encapsulated by a South African based participant in response to whether they are contemplating emigration:

*“I haven’t made the decision to leave. I have made the decision to make plans.”* P16

Emigration plan emerged as a major theme with 4 sub-themes namely “Emigration intention”, “Already emigrated out of SA”, “Willing but not able to emigrate” and “Planned duration of emigration”. For emigration intention in the SA-based participants, they generally had one of two responses: either contemplating emigration with concrete plans or no concrete plans.

Amongst the participants who had already emigrated, the destination countries included the United Kingdom of Great Britain, New Zealand, Canada, Australia, and Ireland. A few participants expressed their willingness to emigrate, which was hampered by issues such as job security, entry examinations in the destination country, security concerns, weather conditions and family structure and support.

Concerning the planned duration of emigration, participants who emigrated or who intended to emigrate viewed it to be a permanent move instead of a short-term plan, because emigration takes a lot of planning and consideration.

### 4.4. Factors Influencing Emigration Plan

In response to questions on factors influencing the emigration plan, eight sub-themes emerged, which could further be categorized into push and pull factors. Most participants who had already emigrated cited pull factors from the destination country as their main reason, while the majority of SA-based participants focused on push factors as their reasons for wanting to leave.

For example, an SA-based participant cited crime, the economy, and the political instability as reasons to leave, while a participant who had already emigrated cited seeking professional growth and broadening their work experience in the first world country as their main reason.

This is illustrated by the following response from a SA-based participant as to why they are contemplating emigration:

*“The crime in the country, the economic situation in the country, and the political instability. It’s mostly those three factors that started plaguing me.”* P1

whereas one participant who had already emigrated responded:

*“The main reason was just to explore what it’s like working in the first world countries… No absolutely, I didn’t move because of South Africa. I just wanted to develop myself and try and progress academically just to broaden my experience.”* P4

#### 4.4.1. Push Factors: Donor Country Factors Encouraging Emigration

Several themes emerged which fell under this category as shown in Table 2. Regarding the bandwagon effect, participants were prompted to emigrate by seeing many colleagues leaving and were concerned over the consequences of staying and missing the opportunity to leave.

As one participant said:

*“Looking around at the moment in anaesthesia in Cape town, so many people are leaving. So many anaesthetists are leaving but I just feel like ‘am I being stupid by not leaving’ and in a couple of years I would kick myself and say, ‘why didn’t I leave at the time when we could’. And it’s scary like everyone is just leaving and I’m scared to be left behind, and I’m scared but I don’t want to go; it’s not a nice place to be in.”* P19

Uncertainty regarding the introduction and implementation of the National Health Insurance (NHI) prompted some SA-based participants to consider emigration due to job insecurity and concerns regarding access to healthcare.

As noted by the following participant:

*“If NHI would to take over and we won’t be able to access medical care like we do now. I don’t know, it would be probably one of my main reasons for wanting to emigrate.”* P9

Both emigrants and SA-based participants cited social reasons such as perceived better opportunities abroad for their families and safety from violent crime as reasons for wanting to emigrate.

One participant said:

*“So physical safety; feeling safe in my house, feeling safe if I drive in the car, feeling safe to have children.”* P20

Participants cited poor infrastructure in South Africa such as unstable water and electricity supplies, pothole ridden roads, and a poorly functioning public health system as major reasons for wanting to leave. They expressed that the inadequacy of the health system often left them feeling frustrated and helpless, and the only way out was to leave the public sector and emigrate. The frustrations listed include missing and malfunctioning equipment, drugs, and consumables that are essential in peri-operative health care.

As one participant reminisced about her time working in the public sector in SA:

*“It was constantly a battle of ‘I know what the patient deserves but I’m physically unable to provide the care that the patients deserve’ because for example we don’t have syringes or we don’t have the needles or surgeries were cancelled because they didn’t order the right set. Or the autoclave is broken or ‘no we can’t do the elective procedures because the temperature in theatre is at twenty eight (28) degrees.”* P8

A few participants mentioned corruption and poor governance as important reasons to emigrate, citing particular concerns over the misuse of taxes and government employee pension contributions. Many participants also cited the deteriorating South African economy, particularly the weak currency and political instability as reasons for wanting to leave SA.

The SA-based anaesthetists working in the public health sector cited feeling unappreciated and not valued as other reasons for wanting to emigrate. However, some participants expressed preference for public over private sector practice, for several reasons, including academia. These participants preferring the public sector expressed an inclination to move abroad if they decided to leave the public sector. Some participants cited race-related issues about ineligibility for certain jobs due to race and concern of potential racial discrimination against their children for selection to attend good schools and universities. Conversely, a black participant felt that race was perceived as the only reason for career progression among black professionals and had emigrated to escape the narrative undermining black progress by attributing it to affirmative action.

One participant expressed the following:

*“…and when you progress as a black woman, it is because you are black. I remember feeling very strongly against that at the time because I thought I was achieving a lot of things regardless of whether I was a black woman or not. But I thought it was always being overshadowed by my race, that people thought it must be easier for certain groups of people because we are the demographics that is supposed to be progressing, helped and chosen.”* P4

The high economic cost of practising in private was cited by some participants. They highlighted the need to work extremely hard to make a decent living due to high income tax bills. They also highlighted the progressive saturation of private practice and the scarcity of public sector posts due to older anaesthetists unwilling to move on. These factors were seen as important push factors.

A participant shared the following regarding migration to private practice:

*“…private was becoming not an easy place to infiltrate, as I was hearing from senior colleagues. As a result, people who ordinarily would have moved on from the public sector and opened up consultant posts were just sitting in those posts, and there weren’t that many posts.”* P21

#### 4.4.2. Pull Factors: Destination Country Factors That Encourage Immigration

The country factors attracting immigration are shown in Table 2.

Participants valued the visible tax spent on public facilities such as hospitals and roads. They also valued flexible working hours, including appropriately compensated overtime, and a better work–life balance. They also mentioned that they felt more protected and appreciated as an employee working abroad, compared to working in the public sector in SA.

As narrated by the following participant:

*“What I like about the UK is there is a lot more...you feel quite protected by the system. So for example, if you get sick and you are meant to work night shift, you are not going to be expected to find someone to do the night shift as is often the case in South Africa. You are just sick and they will cover you, they will pay someone to cover your shift. I think in South Africa it’s a lot of responsibility towards being there. Even if you are not well.”* P6

Some participants felt that SA did not offer as many options for academic career progression compared to countries abroad, and others valued the potential to acquire internationally accredited qualifications.

One participant shared that specialising in Ireland had the advantage of an internationally recognised qualification:

*“So if I become a Fellow of College of Anaesthetists of Ireland, that is recognized in a lot of countries so it actually opens more doors in terms of if we were to move again, we could easily go somewhere else you know now I would again be able to practice without having to re-qualify all over again.”* P8

Regarding race-related issues in the destination country, participants preferred countries perceived as racially accepting and open as shared by the following participant:

*“I think the biggest one for me is race, I need to be in a place where my family will be comfortable. I think New Zealand is an option due to that reason, it was more for the race factor. So it depends on how accepting or welcoming the country is to different races.”* P1

#### 4.4.3. Potential Factors That Would Influence Decision Not to Emigrate

This theme emerged in response to the South African based participants being asked, what needed to change to dissuade them from emigrating. Six sub-themes emerged from the analysis of their responses and are listed in Table 2. These responses were mostly solutions to the South African push factors previously cited by participants for wanting to emigrate. Some participants cited the availability of anaesthetic sub-specialities and research career options as one way of encouraging these professionals to stay.

As one participant said:

*“…So, the only type of sub-specialty we can do here is ICU. It is also quite hard to get in sometimes. So, I think if we offer more subspecialties especially for young or junior consultants it would be great. Or additional sort of research courses or whatever it is where you can actually sort of grow in a different way.”* P12

Strong motivators cited for staying in SA were patriotism and pride for the country and strong family ties.

As one participant narrated:

*“Things like family, things like being South African, being proud South African; growing up in South Africa and wanting to change and being part of a change for a better South Africa I think that’s reasons why one would stay.”* P22

Other possibilities cited were the benefits of improved collaboration between the public and private sectors for anaesthetists. Participants felt that working in both the public and private sectors would allow them to experience the best aspects of both sectors: a well-resourced workplace and good renumeration in private, and the collegial support in the public sector.

One participant said:

*“I would think it would be a bigger balance or cooperation between private and public. I really love working in the public sector because it gives me access to a world of knowledge. I feel supported. You know in the sense of having colleagues and those kinds of things but it is very consuming and there is not much money or the money isn’t as much as you could get in private. Sometimes you do feel taken advantage of with regard to overtime so if that had to change, I would have a better work life balance or control over my life, my time…I would like to be in a space where I could work fifty private, fifty in government. And I think something like that would actually allow one to be more giving in the public sector, you would be more enthusiastic.”* P18

## 5. Discussion

### 5.1. Emigration Plan

In this study, all the participants living in South Africa were considering emigrating. This is significantly higher than previous studies which found between 58% and 66.6% of South African healthcare workers considering leaving South Africa [5]. This finding suggests that emigration poses a potential threat to the South African anaesthetics workforce, which is compounded by the fact that participants viewed their emigration as permanent.

The study also suggests that the participants often contemplate emigration without concrete plans, and they may not take the next step to emigrate, due to obstacles such as entrance exams, pregnancy, and worries about job and physical security in the destination country. Thus, 39% of participants in this study had already emigrated; however, there are no data on South African health worker emigration to compare with. New Zealand, Australia, the UK, Ireland, and Canada were popular choices in this study, similarly to previous studies [5,10,11]. The United States of America was not a favoureddestination country in this study, possibly because the South African anaesthesiology fellowship examinations are not recognized by the American Board of Medical Specialists.

### 5.2. Factors Influencing Emigration Plan

This study showed that factors such as poor infrastructure and resources, corruption, lack of appreciation for the services provided, environmental and socio-economic policy concerns, and safety concerns for family members are important drivers of emigration among the South African anaesthesia workforce. These factors are similar to previous studies [3,5,10]. This study coincided with the violent protests and riots that took place in Durban and Johannesburg in July 2021. These unfortunate events appear to have increased participants’ desire to leave the country due to the socio-economic instability and security concerns that SA faces.

Private practice used to be an attractive and practical alternative to working in the public sector. Migration to the private sector mitigated the push factors often associated with public sector work, such as poor infrastructure and resources, corruption, and a lack of appreciation for the services provided, as noted by Bidwell et al. [3] and Van der Spuy et al. [12]. In contrast, this study found that private practice is no longer as appealing an alternative as it once was due to private sector saturation, particularly in large urban centres such as Cape Town and Johannesburg. In addition, participants expressed that working privately comes with the burden of having to work longer hours to offset high income taxes. In addition, participants from both the public and private sectors expressed their concerns about the implementation of the National Health Insurance (NHI), a universal health coverage system, and how the NHI will affect not only their jobs but also access to healthcare. Concerns about the NHI have not previously been identified as a push factor in studies of SA healthcare worker emigration. This is most likely because the NHI is a more recent development. The Green Paper on the NHI was published on 11 August 2011, with the policy aiming to ensure universal access to adequate, efficient, and quality health services [13].

Unfortunately, the participants’ concerns are not unwarranted. Many authors have highlighted the potential shortcomings in implementing the NHI in SA, including the lack of clear strategies and timeframes for implementation, and the misalignment between Human Resource for Health policy and NHI policy [14,15]. An example would be Ghana, which successfully introduced NHI in 2003. However, they faced challenges such as poor coverage, poor quality of care, corruption and ineffective governance, poor stakeholder participation, lack of clarity about concepts in politics, strong political clout, and poor funding [16].

Another insight from this study is how affirmative action plays an important role in the emigration of healthcare workers, particularly anaesthetists. Affirmative action is otherwise commonly cited in the literature as a reason for emigration among other sectors in South Africa [17]. This finding can be explained by the fact that the former apartheid regime in South Africa systematically excluded black people from undergraduate and postgraduate courses in health sciences. In an effort to address the resulting racial and gender inequalities in specialiststraining, policies have been adopted that encourage affirmative action for the recruitment and enrolmentof black doctorsfor specialist training. These guidelines are relevant as legal remedies in South Africa, but they are generally perceived as discriminatory against certain groups within society. Ironically, the group within society that is thought to benefit from these policies also bears the brunt of it, if they are seen as unjust. It is clear from these findings that affirmative action is a complex and sensitive issue within the anaesthesia fraternity in SA currently.

Regarding factors driving immigration to specific destination countries, the results indicate that South African anaesthesiologists are motivated to move to those countries based on a range of factors. These factors include better financial and physical security, better employment opportunities and working conditions, career growth, and the opportunity for international accreditation and recognition of their qualifications. These results are consistent with previous results from Awases et al. [5], Bidwell et al. [3] and Van der Spuy et al. [12]. The study results also indicate that the potential for adventure, travel and living in a society free from racial discrimination were also seen as factors driving immigration to these destination countries.

The findings of the study also indicate that for the emigration group of anaesthesiologists, these pull factors from the destination country played a more important role in the decision to emigrate. They left SA because of what was offered to them in the destination country, not because of what was wrong with SA. This result is in contrast to a previous study by Bezuidenhout et al. [7] in which they examined the reasons for the brain drain of South African doctors. Their results suggest that donor country push factors often play a more important role than destination country pull factors. These results show two things. Firstly, the perceived pro-immigration factors from the destination country often contrast with the perceived pro-emigration factors from the home country. Second, these perceived factors that encourage emigration seem to carry more weight in the initial motivation to emigrate, but ultimately, what the destination country offers also plays a major role in the final decision to emigrate.

### 5.3. Potential Factors That Would Influence the Decision Not to Emigrate

The study results on potential retention strategies proposed by the South African-based participants demonstrate an association between which factors they perceive to promote emigration from South Africa and what needs to be done to try and retain them in SA. The retention strategies are often solutions to the perceived push factors from SA or the implementation of pull factors from their desired destination country.

Unfortunately, factors such as improving political leadership, resources and infrastructure, improved accountability and improved security are extremely complex in South Africa. Finding a way to improve them requires extensive collaboration between the national government and private stakeholders. However, from a policy perspective, factors such as increasing the availability of anaesthesia specialties and improving collaboration between the public and private sectors are more viable retention strategies for anaesthesiologists to implement.

Finally, the study found that patriotism and family ties in SA were important motivators for anaesthesiologists to consider staying in SA. This result is significant, as these two factors are the only two stay/stick factors identified in this study. A stay/stick factor is a term used to describe those factors that motivate individuals to stay in their home country. However, it remains unclear whether these stick/stay factors will be enough to outweigh their desire to leave.

## 6. Strengths and Limitations

### 6.1. Strength

The strength of this study is that it is a first of its kind to shed light on the brain drain in the field and stimulate discussion. It was cost-effective, and it met its original study objectives. It therefore paves the way for future studies examining migration in the South African anaesthetics workforce.

### 6.2. Limitations

1. The concentration of the study population around a specific age group. The most likely explanation for this is the use of the snowball method to sample participants, since it relies on recommendations from other participants. This would indicate that this group of participants was often more enthusiastic about volunteering.
2. An inherent limitation of qualitative research is the possible participant response bias which is created by researcher presence during the data collection process. The researcher’s own history of emigration could have created this bias.
3. The findings from this qualitative study cannot be generalized to the whole anaesthetic workforce in SA due to the small sample size and therefore cannot be taken to represent all the views on emigration.
4. The researcher could have interviewed key stakeholders within the South African Anaesthesia Fraternity such as SASA, CMSA and HPCSA to obtain their perspective.

## 7. Conclusions

The study found that all the SA-based participants were contemplating emigration, while participants who had already emigrated were not considering returning to SA in the near future. The consideration of emigration was often motivated by push factors related to unsatisfactory living and working conditions in South Africa, such as a high level of crime and corruption and overall poor resources and infrastructure in the country. Poor working conditions in the public sector werecompounded by the feeling of not being valued as an employee.

Destination countries were selected based on their pull factors, which often offset the South African push factors. The pull factors played a crucial role in the decision to emigrate. The decision to emigrate was difficult and emotional and often permanent once made. However, between the period of reflection and the decision to emigrate, there was a possibility of a decision to stay. Factors motivating South African anaesthesiologists to stay include hope and belief, mixed with patriotism, and that things may eventually get better.

## 8. Recommendations

### 8.1. Health Professions Council of South Africa (HPCSA)

The results of this study will be shared with the policy makers, and recommendations on migration policy in South Africa will be made. One of these key stakeholders is the HPCSA, a statutory body that regulates healthcare professionals in South Africa. HPCSA could play an important role in monitoring the emigration statistics of South Africa. Currently, health practitioners can register with the Council whether they are actively practicing in South Africa or abroad. The recommendation would be that HPCSA encourageshealth practitioners to disclose their residency and emigration status, to track where South African health practitioners are based.

### 8.2. South African Society of Anaesthetists (SASA)

An important stakeholder is SASA, an association of public and private sector anaesthesiologists and anaesthetists in South Africa. SASA consists of five business units attending to issues affecting anaesthetists in the Public Sector, Private Practice, Education, Practice Regulations and Special Interest Groups (subspecialities). SASA is therefore key to strengthening the current collaboration between the public and private sectors, and advocating for education and professional growth. SASA will also be key in the negotiation of the position of Anaesthetists in the NHI dispensation.

### 8.3. Colleges of Medicine of South Africa (CMSA) and Medical Universities Training Anaesthesiologists

The CMSA oversees the board examinations for postgraduate specialization and sub-specialisation in SA, while the universities and attached hospitals provide the training platforms Their role will be to expand the available sub-specialties and Fellowshipsin SA.

### 8.4. Future Research Opportunities

A follow-up study of the South African-based participants can be conducted to examine whether they have migrated and the reasons for their decision, which can build on the results of this study. Another follow-up study may be conducted in expatriate participants to examine whether they returned to SA, and the reasons for their decision.

## Figures and Tables

**Table 1:** Socioeconomic demographics of participants (n = 23).

Sociodemographic Variable	Frequency	Percentage
**Age**		
20–30	2	8.7
31–40	16	69
41–50	4	17.4
51–60	1	4.3
**Gender**		
Female	17	74
Male	6	26
**Race**		
White	14	61
Black	6	26
Indian	2	8.7
Coloured	1	4.3
**Highest Anaesthetic Qualification**		
DA	5	21.7
FCA and/or MMed	17	74
PhD	1	4.3
**Years of Experience**		
1–5	2	8.7
6–10	13	56.6
11–15	5	21.7
>16	3	13

**Table 2:** Emerging themes and sub-themes.

Emerging Themes	Sub-Themes
Emigration Plan	▪ Emigration intention, ▪ Already emigrated out of SA ▪ Willing but not able to emigrate ▪ Planned duration of emigration
Factors Influencing Emigration Plan	**Push factors** Band wagon effect National Health Insurance Social and family reasons Poor infrastructure and resources Corruption in SA Non-appreciation of services delivered Race-related issues Economic cost of practicing in private Environment and socioeconomic-political concerns Concerns on security and safety of family members **Pull factors** Socioeconomic development of the country emigrated to Better working conditions Security in the country emigrated to Better job opportunities Financial security Exposure, pleasure, and new environment Professional growth Accreditation and international recognition Race related issues
Potential Factors That Would Influence Decision Not To Emigrate	Improved political governance, resources, and infrastructure Accountability and transparency with public resources Improved security and safety Availability of anaesthetic speciality varieties Patriotism and family Improved collaboration between the public and private sector for anaesthetists

## Data Availability

The interviews transcribed and analysed for this study are not publicly available. However, this could be shared by the authors upon reasonable request and with permission of Walter Sisulu University.
